# The impact mechanism of ownership change on university innovation

**DOI:** 10.1038/s41598-023-50482-w

**Published:** 2023-12-27

**Authors:** Panjun Gao, Xing Li, Guiyang Zhang, Yong Qi

**Affiliations:** 1 School of Intellectual Property, Nanjing University of Science and Technology, Nanjing, China; 2 School of Economics and Management, Nanjing University of Science and Technology, Nanjing, China

**Keywords:** Statistics, Scientific data

## Abstract

The mixed-ownership reform of job invention achievements (MOJIA) is an important exploration of China’s sound long-term incentive mechanism for transforming job-related inventions. Based on the data of MOJIA pilot universities and regions from 2012 to 2022, this paper analyzes the relationship and mechanism between MOJIA and university innovation (UI) in China by combining resource dependence theory and institutional theory. The study found that MOJIA has a promotive effect on UI. The findings continue to hold after using parallel trend tests, lagged regressions, alternative UI measures, endogeneity control, and placebo tests. MOJIA can enhance the technology achievement marketability in the regions where universities are located. Moreover, MOJIA can facilitate the technological achievements marketability by improving UI. Heterogeneity analysis found that the lower the administrative level and the university’s social reputation, the stronger the promotion effect of MOJIA on UI. The research in this paper provides implications for further improving MOJIA.

## Introduction

The knowledge achievements produced by academic scientists (e.g., university teachers and researchers) have significantly impacted technological progress and economic growth. Academic scientists can increase innovation by expanding the scientific base and generating inventions for industrial applications^1^. In the past decade, China's R&D investment has been growing at an annual rate of more than 20% per year, trailing only the United States^2^. According to the Incopat database, Chinese universities have been ranked first in annual invention patent applications in the last decade, surpassing the combined number of patent applications from US, Japanese, Korean, and UK universities^3^. Regarding the patent transfer rate, China's average annual transfer rate has been below 5% for a long time. In comparison, the average annual transfer rate in the US is higher than 54%. As for the growth rate of patent transfer, universities in the UK are the highest at 4.48%, followed by Japan at 3.41% and Korea at 2.99%, while the growth rate of Chinese universities is only 0.19%^3^. It can be seen that Chinese universities have made significant achievements in the field of basic research, but there are still some problems. One typical issue is the allocation of job inventions ownership. Job invention is a form of job scientific and technological achievements under the scope of patent law. Namely, the invention and creation accomplished by carrying out the unit's tasks or using the unit's material and technological conditions^4^. The main problem at present is that universities own ownership, resulting in job inventors' low motivation, unreasonable reward payment mechanisms, and an imperfect system of laws and regulations. This has limited to some extent the output of high-quality innovation in universities, resulting in a paradox between the dazzling innovation output of universities and the lack of university job inventors' innovation motivation.

Scholars have long argued that ownership is critical to corporate strategy and competitiveness^5,6^. Many papers have validated this view and examined the relationship between ownership and innovation, and firm performance. Research on ownership and innovation has focussed on the impact of the identity or distribution of ownership. In terms of ownership structure, Choi et al.^7^ argue that state and institutional ownership positively affected innovation performance but with a lag. Chen et al.^8^ identified ownership diversity and concentration as essential influences on firm innovation. Based on the efficiency and institutional logic perspectives, Zhou et al.^9^ argued that minority state ownership is the optimal structure for innovation development. Regarding the relationship between ownership and firm performance, scholars have argued that employee ownership increases their efficiency and motivation, increasing firm productivity^10–12^. Moreover, higher co-ownership leads to higher product market liquidity, which helps firms with product development and investment^13^. Although these studies thoroughly discuss the relationship between ownership, innovation, and performance, they do not address the inventions ownership by university researchers. Therefore, we do not have a clear picture of the impact caused by changes in the ownership of university researchers' inventions.

Scholars have focused on the ownership and transfer of university inventions in university-level studies. For example, Sterzi^1^ argues that universities have greater autonomy and, therefore, more patents for countries without specific legislation on academic patents. In Europe, where professorial privilege prevails, the ownership of functional inventions is differentiated according to the nature of the employee. In this context, university professors hold the patent rights of their research results, i.e., the university inventor owns the invention in the office. However, for researchers employed in private companies, the patent rights of the invention in the office belong to the employer^14^. Thursby et al.^15^ found that assignment to inventor-related start-ups is less likely the higher the share of revenue inventors receive from university-licensed patents. Von et al.^16^ examined ownership patterns of German university-invented patents before and after the abolition of the “professors' privilege” in 2002 to explore how the legal change affected patenting activities. After the reform, they found no significant patent increase and a gradual shift from individually- owned and company-owned to university-owned patents. In addition, some scholars have studied the factors influencing the transformation of university job invention results, such as university innovation intermediaries^17^, technology holding companies^18^, and university industrial cooperative research centers^19^, quality of research results^20^, and university strategic choices^21^.

While the above studies have significantly improved existing knowledge, little attention has been paid to how university researchers' ownership change affect university innovation (UI). In the past few years, many universities have gradually carried out the mixed-ownership reform of job invention achievements (MOJIA), and the job inventors (researchers) have a certain degree of the invention achievements ownership. In 2016, for the first time in Sichuan Province, there was a mixed-ownership reform of job invention achievements (MOJIA). The rights of scientific and technological achievements were allocated to scientific researchers, marking a breakthrough in the allocation of scientific and technological achievements' rights in China's universities. Mixed-ownership reform of job invention achievements (MOJIA) refers to changing the university patent application right or patent ownership, shifting intellectual property rights from unit ownership to unit and individual common ownership, and changing the incentive of post-facto income into the incentive of prior property rights. It confirms the ownership status of scientific and technological innovation achievements by researchers, thus increasing their motivation to carry out scientific and technological R&D and transformation work. MOJIA provides an ideal research environment to reveal the impact of ownership changes. This is because a university's innovation ability may change dramatically with ownership change of researchers' inventions.

With the implementation of MOJIA, several vital issues require attention. They are as follows. Can MOJIA enhance UI? Through what channels does this occur? What is the role of university characteristics in this relationship? Considering this, in most countries, universities are the central bodies performing basic research, and the government allocates resources and policymakers that can influence the universities' innovative behavior^22^. This means that the analysis of UI needs to consider the different goals and roles of government and universities^23^. To this end, we take China as our context by combining resource dependence theory and institutional theory, matching university and regional level data to analyze the potential relationships and mechanisms between MOJIA and UI.

Compared to existing studies, our study makes several contributions. First, existing research tends to link innovation to specific ownership. For example, institutional ownership^24^, family ownership^25^, and state or foreign ownership^7,9,26^. Based on existing studies, we analyze the impact of job inventors’ (university researcher) ownership change on university innovation by focusing on changes in job inventors’ ownership. And then examine the relationship between ownership and university innovation from a dynamic perspective. The study found that MOJIA can promote UI.

Second, resource dependence theory mainly studies the dependent relationships between enterprises. However, UI relies on technological, intellectual, and government financial and policy resources^27^. By integrating resource dependence theory and institutional theory, we established a unified analytical framework to test the relationship between ownership institution (MOJIA) and UI. We explored the boundary conditions involved in university characteristics based on China’s unique institutional status quo. To some extent, our study exceeds previous studies that have analyzed resource dependence theory mainly from an intra-firm perspective^9,28^. In addition, further analysis of institutional factors with Chinese characteristics, such as the role of intellectual property protection (formal institution), administrative rank, and social prestige of universities (informal institution), can deepen the understanding of UI issues.

Third, our paper also makes several empirical contributions. We use MOJIA as a natural experiment and DID to determine the impact of changes in researchers’ invention ownership on UI. To the best of our knowledge, this is one of the first studies to examine the relationship between ownership and UI, and one of the first to flesh out the potential mechanisms of researchers’ invention privatization. We created a unique dataset to study the innovative behavior of these universities by basing our study on data from the Compilation of Scientific and Technical Statistics of Higher Education, PatSnapy, and the China Statistical Yearbook published by the Chinese Ministry of Education. Therefore, this study provides literature support for the enhanced innovation capacity in China and other emerging economies, filling a gap in the existing empirical research.

The article proceeds as follows. Section “Institutional background and theoretical analysis” covers the institutional background and research hypothesis. Section “Research design” covers the model design, describe the variables, and the sample data. Section “Results” presents the benchmark regression, and robustness tests. Section “Further analysis” provides the heterogeneity analysis, the moderating effect of intellectual property protection, and the economic consequence of UI. Section “Discussion and Conclusions” provides the discussion, managerial and policy implications, and limitation.

## Institutional background and theoretical analysis

### Institutional background

In 2015, based on the preliminary exploration of Southwest Jiaotong University, Sichuan Province issued the Decision of the CPC Sichuan Provincial Committee on Comprehensive Innovation Reform to Drive Transformation and Development, proposing to "carry out a pilot mixed ownership system of scientific and technological achievements, and clarify that scientific researchers and their affiliated units are co-owners of scientific and technological achievements". It is determined that 20 universities and research institutes in Sichuan undertake the critical task of systematically promoting the comprehensive innovation reform trial, and universities and job inventors can share the ownership of invention results, which "awakens" many "dormant" job invention results. Immediately after that, in 2018, the pilot of mixed ownership reform of job invention achievements is intended to be expanded in 45 universities, institutes, and enterprises in Sichuan Province.

In May 2020, the Ministry of Science and Technology (MOST) and others jointly issued a notice on the "Pilot Implementation Plan for Granting Researchers the Right to Ownership or Long-term Use of Scientific and Technological Achievements on Duty", making a substantial step forward in deepening the reform of the right to use, dispose of and benefit from scientific and technological achievements. Moreover, 41 pilot units were announced that year, covering Beijing, Shanghai, Jiangsu, and other places, with a gradually expanding scope.

In recent years, as the state has issued several policy documents to promote the MOJIA continuously, the reform has become the focus of discussion in the field of job invention. According to the provisions of the Pilot Implementation Plan for Granting Scientific and Technological Achievements on Duty to Scientific Researchers or Long-term Use Rights, "the ownership of scientific and technological achievements on duty completed by scientific researchers in institutions of higher education and scientific research institutions established by the state belongs to the unit", and the unit can transfer the ownership of scientific and technological achievements on duty to the accomplished person with a certain proportion under this premise and its common ownership. This "mixed ownership" model of job invention has the characteristics of both "employees" and "employers", which can balance the interests of both units and inventors.

### Theoretical analysis

Due to environmental uncertainties and resource constraints, it is difficult for an organization to have all the resources needed for R&D activities. Therefore, it will form resource-dependency relationships with external stakeholders. According to resource dependence theory, organizations must interact with external subjects to obtain the required resources^29^. The innovative activities of universities require human and knowledge resources, a stable business environment, and a sound intellectual property protection system provided by the government. Universities also depend on government financial support for their operations and research funding for researchers^30^. In addition, universities have a threefold mission of teaching, research, and human resource development, and there are various disputes over the ownership of results. Therefore, it is necessary to consider the homogeneity of the system. The system will keep the subjects' behavior consistent through forced isomorphism, imitative, and normative isomorphism^31^. As a policy-maker and resource allocator, the Chinese government has a large amount of exclusive information and scarce resources that are critical to the survival and operation of organizations. Different policy norms and resource allocation schemes will guide the R&D behavior of enterprises and universities and play the dual roles of supporting hand and intervening hand^30^.

MOJIA adopts the strategy of “first share the land, then share the food” by adjusting the structure of property rights among the suitable subjects and giving the researchers the right to apply for and own part of the scientific and technological achievements. Such reforms may introduce a more flexible and equitable profit-sharing mechanism, enabling teachers to benefit directly from their job-related inventions. This direct financial incentive has the potential to encourage teachers to invest more in research and innovation, knowing that their efforts can be directly translated into material rewards^32^. In this way, it helps to promote the sharing of resources and stimulate the innovation enthusiasm of diversified subjects such as universities and enterprises and scientific research units^4^. Second, MOJIA may motivate universities to seek more opportunities for external collaboration. The reform may make universities more willing to cooperate with external organizations, such as commercial companies, through which they can obtain more financial and technical support and make effective commercial use of their job inventions. This kind of external cooperation can bring direct economic benefits to universities and introduce new technologies and ideas to promote innovative activities further^29^. Finally, MOJIA may change the resource dependency structure of universities. Under the traditional public ownership model, colleges and universities rely primarily on government support. However, MOJIA may cause colleges and universities to seek support from other sources, such as enterprises and the public. Such a change could lead to more diversified access to university resources, increasing their stability and resilience in the face of environmental change. At the same time, more comprehensive resource dependence may also lead to more innovative opportunities for universities^33^.

In addition, China’s political system is characterized by a high degree of centralization. Both basic research in universities and innovation activities in enterprises are closely related to institutional norms and government actions. Hong and Su^34^ argue that China’s central and local governments represent two sources of institutional power that can encourage exchange and cooperation between universities and enterprises and are not affected by geographical distance. In addition, the main purpose of MOJIA is to promote innovation and development by transforming scientific and technological achievements. The University’s MOJIA policy attempts to realize further shared property rights by loosening the policy. The value of researchers' intellectual labor is recognized. The loosening of property rights management in universities will be conducive to fully sharing results and resources, and the “triple helix” model of industry-university-research cooperation^35^ will be further deepened. At the same time, MOJIA stimulates scientific and technological cooperation and innovation through the effective allocation of resources, creating a favorable environment for scientific and technological innovation and improving the output and efficiency of university scientific and technological innovation. Logically, the coming hypothesis is proposed.

#### Hypothesis 1

The MOJIA is positively affects UI.

Innovation has the characteristics of externality and quasi-public goods. Therefore, it requires a sound legal protection environment or mechanisms that can reduce the risk of information disclosure and protect the rights of innovative subjects^36^. In particular, IPP is an important contextual factor for innovation activities, directly affecting an organization's ability to control and exploit its intellectual property resources effectively. When the level of IPP is high, universities are more likely to derive satisfactory returns from their research and development activities. Strong IPP mechanisms prevent other organizations or individuals from illegally using or copying their inventions and technologies, thus ensuring that universities and faculty can reap the financial benefits they deserve^37^. At the same time, researchers may be more motivated to engage in innovative activities because of IPP, knowing their efforts will not be easily plagiarized. These effects will likely motivate universities to increase their investment in innovation activities, thereby increasing innovation output^38^.

In addition, IPP assists organizations in deriving value from innovation, securing return on investment, and maintaining a long-term competitive advantage over rivals^39^. MOJIA gives the university patent inventor a certain level of ownership and, at higher levels of IPP, better guarantees that the patent inventor will benefit from the transfer or licensing of the patented technology. The patented technology of the university's scientific and technological achievements is promoted to be applied in commercialization and marketization to realize the transformation to actual productivity. Not only that, when IPP is high, it is more conducive to accelerating the diffusion of technological information, reducing duplication of investment by universities or enterprises, and allowing other innovators to stand on the shoulders of the giants to develop new technologies and devices^40–42^. This accelerates technological innovation and enhances competition in technology markets. Based on the above analysis, we propose the following hypothesis.

#### Hypothesis 2

The higher the IPP, the stronger the contribution of MOJIA to UI.

It is clear from the above analysis that MOJIA helps to promote UI. According to resource dependence theory, universities usually depend on multiple external resources in realizing innovation and technology transformation^43^. These resources include government funding, technical and financial support from industrial partners, transfer of patented technologies, and outstanding talents. Also, with their extensive social connections and numerous platforms for knowledge innovation, universities are vital nodes for all types of knowledge flows and have significant knowledge spillover effects^44^. Universities become powerful engines of innovation when they strive to push the boundaries of knowledge through incubation, entrepreneurship, and commercialization^45^.

With the implementation of MOJIA, researchers have gained some invention ownership, and the research capacity and university innovation output have increased, resulting in more unique resources and competitive advantages in technology development and innovation. With increased innovation capacity, universities can reduce over-reliance on external resources. For example, under their innovative capacity, universities can rely more on independent research and development and reduce the need for external technology transfer, which reduces the cost and risk of technology transformation. At the same time, increased innovation capacity makes universities more attractive and better able to work with external partners such as industry, investors, and government. These partners can provide universities with more resource support and help them transform their technological achievements into marketable products. In short, as an organization with a broad range of resources, universities can better transform innovations into marketable products through cooperation with enterprises, industrial parks, research institutes, and the government. This resource integration helps break down barriers to innovation and accelerate the process of technology achievements marketability. Considering the above arguments, we believe that MOJIA can facilitate the technology achievements marketability by improving UI.

#### Hypothesis 3

MOJIA can facilitate the technology achievements marketability by improving UI.

**Figure 1:**
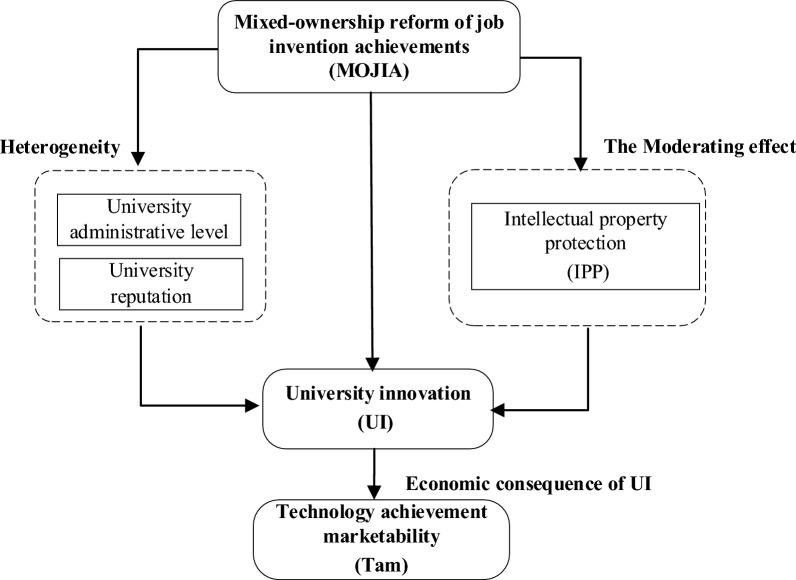
Theoretical framework.

Based on the hypotheses in this study and the main research components, a theoretical framework figure was created, as shown in Fig. 1. Among them, MOJIA is the core explanatory variable, University innovation (UI) is the dependent variable, Intellectual property protection (IPP) is the moderating variable, and Technology achievement marketability (Tam) is the economic consequence. And we analyze the heterogeneity in terms of university administrative level and reputation.

## Research design

### Model design

The impact of MOJIA on UI may face endogenous problems. First, unobservable factors such as the university's history, culture, and other individual traits that do not change over time may affect MOJIA and UI activities. Second, there may be a reverse causal relationship between the mixed-ownership reform of job invention achievements (MOJIA) and UI. The amount of university innovation achievements may also influence the pilot policy. To accurately assess the impact of MOJIA on UI, we used MOJIA as a quasi-natural experiment. Previous studies have shown that the DID model can effectively assess quasi-natural experiments^46,47^. Therefore, this study will construct two-way fixed effects multi-time point DID based on the MOJIA lists of universities in 2016, 2018, and 2020. The estimating equations are as follows.

1 $$UI_{it} = \alpha_{0} + \alpha_{1} \times did_{it} + \alpha_{2} \times Controls_{it} + u_{i} + v_{t} + \varepsilon_{it}$$

where *UI* represents the dependent variable, that is, university innovation (*UI*), *did* describes MOJIA. Suppose the university’s location is the pilot university during the sample period, and the observation time is after the selected year. In that case, the value of *did* is 1; otherwise, it is 0. The coefficient *α*_*1*_ measures the net effect of MOJIA on UI. If *α*_*1*_ > 0, it indicates that the MOJIA promotes UI. *Controls* represent each control variable, *u* and *v* represent the fixed effect of university and year, respectively. $\varepsilon$ is the random error term.

### Variable measurement

#### Dependent variables

University innovation (UI) is the dependent variable of this paper. Universities play an essential role in the national innovation system, and university innovation is an essential element of science and technology management. As a significant output of innovation activities, universities’ patents can reflect the level of university technology innovation. Specifically, we construct a patent-based measure of innovation output. This measure is the total number of patent applications filed by universities in a given year that is granted. Moreover, we match the patent application year (not the grant year) with other data, as the superiority of the former in reflecting the timing of innovation^48–50^. To reduce the effect caused by heteroskedasticity, we take the natural logarithm of the variable^51^.

Technology achievement marketability (Tam). This paper uses the technology achievements marketization index from China Market Index Database to measure *Tam*. This index initially used the ratio of technology market turnover to the number of local science and technology personnel in each locality as an indicator. However, since the statistics of science and technology personnel in each province are no longer published after 2016, the ratio of technology market turnover to local GDP in each place is used instead after 2016.

#### Explanatory variable (*did*)

We take the mixed-ownership of job invention achievement (MOJIA) as an exogenous shock event for a quasi-natural experiment. Suppose the listed universities are belong to the MOJIA demonstration university, and the data year is after the policy implementation year. In that case, then *did* is assigned a value of 1. Otherwise, the value is 0.

#### Moderating variable (intellectual property protection, *IPP*)

This article refers to the approach of Li et al.^52^ and measures the level of intellectual property protection from four dimensions: Degree of judicial protection, degree of social legalization, awareness of intellectual property protection, and regional economic level. The degree of judicial protection is expressed in terms of the non-infringement rate. When the value is higher, it indicates that IPR protection in the region is more effective. The degree of social legalization is measured by the number of lawyers per 10,000 people in each region, and the higher the ratio, the greater the degree of social legalization in the region^53^. In addition, we use the number of regional universities to measure awareness of IPR protection^54,55^, and GDP per capita to reflect the regional economic level^55^. The main reason is that the public's awareness of intellectual property rights is the basis for implementing intellectual property law. The public's awareness of intellectual property rights is an important factor affecting the intensity of law enforcement. It is generally believed that the higher the education level of the public, the higher their awareness of intellectual property rights^54^. The regional economic development level is closely related to IPR protection. When people have satisfied their physiological and safety needs, they may consider high compliance needs^53^. Therefore, the higher the regional economy level, the higher the IPR protection is likely to be. The specific measurements are shown in Table 1.

**Table 1:** Measurement of intellectual property protection.

Variables	Method of measurement
Degree of judicial protection^56^	1-(Number of patent infringement cases accepted by the region/number of patent authorizations in the current year)
Degree of social legalization^53^	The number of lawyers per 10,000 people in the region divided by 10,000
Awareness of intellectual property protection^55^	The logarithm of the number of inter-provincial universities
Regional economic development level^55^	The regional GDP per capita

#### Control variable

We include a rich set of control variables in our models to mitigate the possibility that our results are driven by omitted variable biases^57^. In line with earlier research, these control variables fall into two broad categories, covering differences at the level of area and the overall university. The control variables at the regional level include government expenditures on science and technology activities (*Gov*) and GDP per capita (*GDP*)^58,59^. The control variables at the university level include R&D expenditure (*Rf*), scientific paper issued (*Paper*), number of subjects (*Subject*), number of awards (*Award*), and R&D personnel (*RDperson*)^3^. We take the natural logarithm of the control variables to reduce the heteroskedasticity problem. Referring to Qing et al.^60^, we constructed Table 2 to provide a comprehensive overview of all defined variables, including their measurements and corresponding definitions. Table 3 reports the results of descriptive statistics for the main variables. This paper tests the variance inflation factor (VIF) for the explanatory and control variables. The results show that the VIF values of all variables are less than 10, indicating no severe multicollinearity problem.

**Table 2:** Variable definition and measurement.

Variables	Name	Symbol	Measurement	Definition
Dependent	University innovation	UI	Total number of patent applications	the natural logarithm of the total number of patent applications
	Technology achievement marketability	Tam	Technology achievements marketization index	Year < 2016: Local technology market turnover/number of scientific and technological personnel Year ≥ 2016: technology market turnover/local GDP
Explanatory	The mixed-ownership of job invention achievement (MOJIA)	did	A quasi-natural experiment	See above
Moderating	Intellectual property protection	IPP	Level of intellectual property protection	See Table 1
Control	Government expenditures on science and technology activities	Gov	Government expenditures on science and technology activities	ln (Gov + 1)
	GDP	GDP	GDP per capita	ln (GDP + 1)
	R&D expenditure	Rf	R&D expenditure of university	ln (Rf + 1)
	Scientific paper issued	Paper	Scientific paper issued of university	ln (Paper + 1)
	Number of subjects	Subject	Number of subjects of university	ln (Subject + 1)
	Number of awards	Award	Number of awards of university	ln (Award + 1)
	R&D personnel	RDperson	Number of university R&D personnel	ln (RDperson + 1)

**Table 3:** Descriptive statistics.

	Variable	N	Mean	SD	p50	Min	Max
Control group	UI	363	6.705	1.103	6.879	2.944	8.500
	did	363	0	0	0	0	0
	IPP	363	0.678	0.0950	0.673	0.458	0.988
	Tam	363	8.780	4.257	8.970	1.130	16.62
	Rf	363	12.71	1.065	12.60	10.28	15.14
	Paper	363	7.560	0.945	7.580	5.147	9.512
	Subject	363	7.155	0.810	7.276	5.198	9.201
	Award	363	2.539	1.063	2.565	0	4.828
	RDperson	363	6.526	0.738	6.599	4.844	8.104
	GDP	363	10.02	0.366	10.02	9.043	10.96
	Gov	363	4.781	0.917	4.688	2.582	7.064
Treatment group	UI	429	6.482	1.430	6.784	1.946	9.014
	did	429	0.399	0.490	0	0	1
	IPP	429	0.718	0.0830	0.682	0.606	0.988
	Tam	429	12.44	3.901	13.41	3.270	22.03
	Rf	429	13.01	1.926	13.06	8.815	16.89
	Paper	429	7.589	1.222	7.519	4.331	9.943
	Subject	429	7.182	1.124	7.212	3.611	9.389
	Award	429	2.368	1.108	2.398	0	4.844
	RDperson	429	6.495	1.029	6.482	2.197	8.919
	GDP	429	11.10	0.496	11.08	10.30	12.16
	Gov	429	7.007	1.833	6.246	3.368	9.325
Total sample	UI	792	6.584	1.294	6.819	1.946	9.014
	did	792	0.216	0.412	0	0	1
	IPP	792	0.699	0.0910	0.679	0.458	0.988
	Tam	792	10.76	4.455	11.08	1.130	22.03
	Rf	792	12.87	1.596	12.84	8.815	16.89
	Paper	792	7.575	1.103	7.538	4.331	9.943
	Subject	792	7.170	0.992	7.256	3.611	9.389
	Award	792	2.446	1.090	2.485	0	4.844
	RDperson	792	6.509	0.907	6.519	2.197	8.919
	GDP	792	10.60	0.699	10.52	9.043	12.16
	Gov	792	5.987	1.853	5.594	2.582	9.325

### Data sources

The data on R&D expenditure, scientific papers issued, subjects, R&D personnel, and awards at the university level from 2012 to 2018 were obtained from the MOE’s Compilation of Scientific and Technical Statistics of Higher Education, while the data from 2019 to 2022 were obtained from the official websites of universities and local government websites. GDP per capita and Intellectual Property Protection data are from the China Statistical Yearbook. The university patent grant data is obtained from PatSnap, and a search is conducted with the sample university as the applicant (patentee), to obtain the number of patents published in the university patent disclosure year. And we use it as the number of patents granted by the university in that year. For missing data at the university level, this paper uses the moving average difference method to supplement them. In the sample selection, in 2016, Sichuan Province launched a pilot policy of MOJIA. In 2018, Sichuan Province expanded the pilot units from 20 to 45, and by 2020, the Ministry of Science and Technology added 40 pilot units. Considering that some universities did not disclose data on R&D expenditures, theses, and dissertations, etc., we finally selected 39 universities as the treatment group. In addition, we selected 33 universities as a control group based on the authority to which the university belongs. For example, in the treatment group, the authority of Sichuan University and Southwest Jiaotong University is the Ministry of Education of the People's Republic of China. Therefore, in the control group, we took Wuhan University and Hunan University, whose authorities are also the Ministry of Education, as the study subjects. Our final data are balanced panel data, including 792 university-year observations from 72 universities from 2012 to 2022.

## Results

### Benchmark regression

Table 4 reports the baseline regression results. Among them, the regression results that only control for time and individual effects are shown in column (1). After gradually adding control variables such as Rf, Paper, Subject, Award, RDperson, GDP, and Gov, the regression results are shown in column (2). Table 4 shows that the explanatory variable’s (*did*) coefficient is significantly positive at the 5% level. The results show that MOJIA positively affects university innovation within the sample. Hypothesis 1 is validated. Therefore, university patents have increased and innovation capabilities have been enhanced.

**Table 4:** Benchmark model results.

	(1)	(2)
	UI	UI
Did	0.1185**	0.2528***
	(2.417)	(4.656)
Rf		0.0134
		(0.433)
Paper		− 0.2257***
		(− 4.031)
Subject		0.0933
		(1.512)
Award		0.0597*
		(1.726)
RDperson		− 0.2144***
		(− 3.314)
GDP		1.9755***
		(4.804)
Gov		0.2226***
		(3.002)
_cons	6.5586***	− 13.6331***
	(391.993)	(− 3.146)
University	Yes	Yes
Year	Yes	Yes
N	792	792
R^2^	0.9289	0.9358
Adj_ R^2^	0.9206	0.9276
F	5.8399	10.2101

*t* statistics in parentheses.

*p < 0.1, **p < 0.05, ***p < 0.01.

The result is similar to the findings of Kenney and Patton^61^. The existing distribution of benefits ignores researchers' creative input, thus affecting university researchers' motivation to innovate. Giving university researchers ownership of results is one of the incentives that motivate them to carry out scientific and technological inventions^62^. From an institutional theory perspective, in MOJIA, university researchers are given greater rights to manage and utilize their research results. Ownership of scientific and technological results by the inventor positively impacts the generation of knowledge spin-offs^61^. If ownership is lost, researchers may be pessimistic about STI efforts^63^. This institutional reform can be understood as an institutional innovation that increases the motivation and incentives of university researchers to innovate by restructuring property rights, which in turn leads to more innovative outputs for the university. Resource dependence theory suggests that the survival and success of an organization depends on its ability to acquire and utilize necessary resources^29^. Under MOJIA, it may be easier for university researchers to commercialize their knowledge resources, translating research results into tangible economic benefits. This means that university researchers have more freedom and choice in accessing resources, which may drive more innovative output.

### Robustness tests

#### Parallel trend test

This paper uses the event analysis method to test the parallel trend^64^. The model is set as follows:

2 $$\begin{aligned} UI_{it} & = \alpha + \beta_{1} \times Treat_{i} \times Before^{4 + }_{it} + \beta_{{2}} \times Treat_{i} \times Before^{3}_{it} + \beta_{{3}} \times Treat_{i} \times Before^{2}_{it} \\ & \quad + \cdots + \beta_{5} \times Treat_{i} \times Current_{it} + \beta_{6} \times Treat_{i} \times After^{1}_{it} \\ & \quad + \cdots \beta_{9} \times Treat_{i} \times After^{4 + }_{it} + \sum {University} + \sum {Year} + \varphi_{it} \\ \end{aligned}$$

where *Before*^*4*+^ is a dummy variable taking a value of one if the observation occurs four or more years before MOJIA policy and zero otherwise. *Before*^*3*^ is a dummy variable taking a value of one if the observation occurs three years before MOJIA policy and zero otherwise. Same for *Before*^*2*^ and *Before*^*1*^. *Current* is the year of the MOJIA policy. *After*^*1*^, *After*^*2*^, and *After*^*3*^ are dummy variables taking a value of one if the observation occurs in the first, second, and third year after MOJIA year, respectively, and zero otherwise. *After*^*4*+^ is a dummy variable equal to one for four or more years after the MOJIA year. The dummy variable for *Before*^*4*+^ is omitted from the regression to avoid the multicollinearity problem. We then include the interaction of the time dummies with the variable for the treated universities (*Treat*). Coefficient *β*_*k*_ indicates the difference between the treatment group and the control group. If the coefficient of *β* is insignificant, and *k* < 5, indicating that the treatment and control groups meet the parallel trend hypothesis. Otherwise, the parallel trend assumption is not satisfied. Table 5 shows the parallel trend test results with UI as the dependent variable. When *k* < 5, the coefficients of *β* are not significant. When k > 5, the coefficient of *β* is significant. This indicates that the difference in the UI level between pilot and non-pilot universities is getting larger, and the parallel trend test is satisfied.

**Table 5:** Parallel trend test.

	(1)
	UI
Treat × Before3	− 0.1081
	(− 1.483)
Treat × Before 2	0.0019
	(0.025)
Treat × Before 1	0.1039
	(1.325)
Treat × Current	0.2110**
	(2.547)
Treat × After1	0.1795**
	(2.363)
Treat × After 2	0.1980**
	(2.512)
Treat × After 3	0.3295***
	(3.175)
Treat × After 4	0.3652***
	(3.413)
Rf	0.0257
	(0.840)
Paper	− 0.2179***
	(− 3.868)
Subject	0.0959
	(1.555)
Award	0.0577*
	(1.675)
RDperson	− 0.2145***
	(− 3.318)
GDP	2.0508***
	(4.214)
Gov	0.2012***
	(2.697)
_cons	− 14.5239***
	(− 2.851)
University	Yes
Year	Yes
N	792
R^2^	0.9366
Adj_R^2^	0.9278
F	6.0683

*t* statistics in parentheses.

*p < 0.1, **p < 0.05, ***p < 0.01.

#### Alternative UI measures

We follow Ji and Yang ^65^ and use the total number of patent granted to measure innovation output. Specifically, we use the number of patent granted in the university and then take the natural logarithm to measure UI. As can be seen in column (1) of Table 6, the coefficient on *did* is significantly positive, suggesting that our conclusions remain valid.

**Table 6:** Lag period regression and alternative UI measures.

	(1)
	UI
did	0.2278***
	(3.361)
Rf	− 0.0097
	(− 0.250)
Paper	− 0.2461***
	(− 3.522)
Subject	0.1029
	(1.336)
Award	0.0729*
	(1.689)
RDperson	− 0.2316***
	(− 2.868)
GDP	2.5652***
	(4.999)
Gov	0.2417***
	(2.612)
_cons	− 20.3779***
	(− 3.768)
University	Yes
Year	Yes
N	792
R^2^	0.9050
Adj_R^2^	0.8930
F	8.9234

*t* statistics in parentheses.

*p < 0.1, **p < 0.05, ***p < 0.01.

#### Endogeneity control

This paper uses MOJIA as a quasi-natural experiment to address some endogeneity issues. However, in reality, the government may pilot the policy in universities with stronger research and development foundations or higher innovation performance. Therefore, there may be endogeneity issues, such as sample self-selection. In order to effectively address the endogeneity problem described above, we will use a Heckman two-stage model for the regression^66^. Specifically, we generate a new variable *Heck_UI* based on the median of the UI, which takes the value of 1 when the UI's value is stronger than its median, and 0 otherwise. The model settings are as follows:

First stage:

3 $$\begin{gathered} Pr(Heck\_UI) = \alpha + \beta_{1} \times Rf_{i,t} + \beta_{{2}} \times Paper_{i,t} + \beta_{{3}} \times Subject_{i,t} + \beta_{{4}} \times Award_{i,t} \hfill \\ + \beta_{{5}} \times RDperson_{i,t} + \beta_{{6}} \times GDP_{i,t} + \beta_{{7}} \times Gov_{i,t} + \sum {University} + \sum {Year} + \varphi_{i,t} \hfill \\ \end{gathered}$$

Second stage:

4 $$UI = \alpha + \beta_{1} \times did_{i,t} + \beta_{{2}} \times Controls_{i,t} + \beta_{{3}} \times \mathop \lambda \limits^{ \wedge } + \sum {University} + \sum {Year} + \varphi_{i,t}$$

where, $\mathop \lambda \limits^{ \wedge }$ is the IMR (Inverse Mills Ration) calculated by model (3). *Controls* are control variables. Table 7 shows the results of the Heckman two-stage regression. We can see from column (2) that the coefficient of *did* is also significantly positive, indicating that the results of this paper are reliable.

**Table 7:** Heckman two-stage regression.

	(1)	(2)
	Heck_UI	UI
did		0.1831**
		(2.086)
IMR		− 0.0734*
		(− 1.711)
Rf	− 0.8441**	− 0.0152
	(− 2.304)	(− 0.246)
Paper	− 1.8426**	− 0.1572*
	(− 2.053)	(− 1.799)
Subject	1.5001**	0.2131**
	(2.082)	(2.396)
Award	− 0.3226	− 0.1087*
	(− 0.751)	(− 1.922)
RDperson	− 0.5603	0.0144
	(− 0.856)	(0.136)
GDP	− 12.4565**	− 0.2353
	(− 2.317)	(− 0.308)
Gov	0.8680	0.4676***
	(1.144)	(4.298)
_cons	143.2187**	6.6978
	(2.441)	(0.803)
University	Yes	Yes
Year	Yes	Yes
N	330	330
R^2^		0.8111
Adi_R^2^		0.7788
F		4.8525

*t* statistics in parentheses.

*p < 0.1, **p < 0.05, ***p < 0.01.

Next, we use the IV method to further address possible endogeneity issues. For IV, we use the lagged one period of MOJIA as an instrumental variable for the regression. The main reason is that the core explanatory variables in the lagged period are highly exogenous while closely related to the core explanatory variables in the current period, which satisfies the requirement of selecting instrumental variables. Relative to the current period, the lagged period's explanatory variables have already occurred and are difficult to be affected by the current period's shocks. Thus, they are uncorrelated with the contemporaneous random disturbance term, which can alleviate the endogeneity problem in the estimation of the benchmark model to some extent^67^. Therefore, we will address possible endogeneity issues, such as omitted variables by combining IV with two-stage least squares. The regression results are shown in Table 8. In the first stage, the regression coefficients of IV are significantly positive, the F-statistic is 127.78, which is greater than the critical value of 10, and the instrumental variable results are robust. In the second stage, the coefficients of the explanatory variables are significantly positive, indicating that the baseline regression results still hold after the endogeneity problem is solved.

**Table 8:** IV method regression results.

	(1)	(2)
	First stage	Second stage
	did	UI
L.did	0.8961***	
	(0.017)	
did		0.3797***
		(0.056)
Rf	0.0261***	0.0132
	(0.009)	(0.029)
Paper	0.0074	− 0.1903***
	(0.017)	(0.052)
Subject	− 0.0120	0.0690
	(0.019)	(0.057)
Award	0.0138	0.0479
	(0.010)	(0.032)
RDperson	0.0048	− 0.2074***
	(0.020)	(0.060)
GDP	− 0.1115	2.2402***
	(0.125)	(0.380)
Gov	− 0.0220	0.3074***
	(0.023)	(0.071)
_cons	0.7225	− 13.3224***
	(1.208)	(3.673)
University	Yes	Yes
Year	Yes	Yes
N	770	770
R^2^	0.942	0.941
F	127.78	

Standard errors in parentheses.

***p < 0.01, **p < 0.05, *p < 0.1.

#### Placebo test

**Figure 2:**
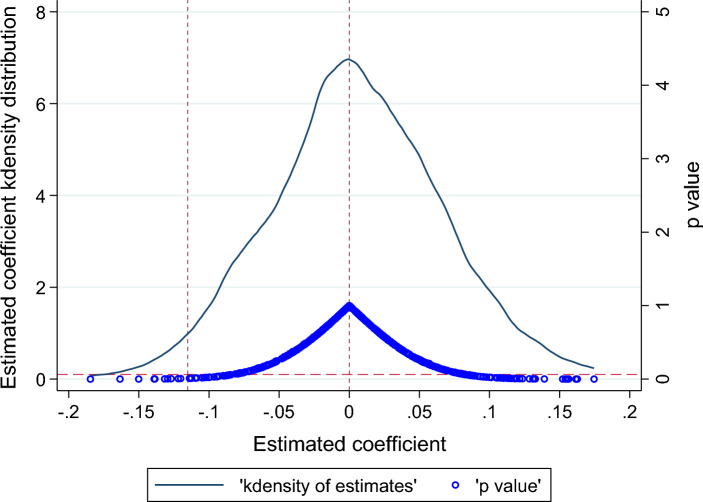
Randomly set the MOJIA time.

We also conducted a further placebo test to validate the conclusions' reliability. We performed a placebo test by randomizing the time of the MOJIA policy. Since the "pseudo" pilot times are randomly generated, MOJIA does not significantly affect UI. The regression coefficients of the "pseudo" treatment variables should be around 0. Otherwise, it indicates that the model of this paper is biased. Based on the above analysis, this paper repeats the stochastic process 500 times for model estimation and plots the kernel density of the estimated coefficients of the "pseudo" MOJIA variables. As shown in Fig. 2, the mean values of the estimated coefficients are close to 0, and most of the p-values are above 0.1. Meanwhile, the estimated coefficients (0.2528) of MOJIA (*did*) are located in the range of small probability events in the kernel density plot. In other words, the boosting effect of MOJIA on UI is not coincidental. Therefore, the findings are reliable and robust.

## Further analysis

### Heterogeneity analysis

#### The heterogeneity analysis of university administrative level

Chinese universities have different administrative levels, such as deputy minister and director general. Administrative rank is a political economy indicator that can reflect the characteristics of the Chinese system. Administrative rank is closely related to resource allocation capacity. The higher the administrative rank, the more resources are at the disposal of the university, which in turn affects the efficiency of resource allocation^68^. Universities at different administrative levels have different on-campus resources and external resources that they can influence. This paper draws on the idea of Yu et al.^69^ that the president's administrative level describes the university administrative level. Specifically, the university president is assigned a value of 1 if he/she is at the deputy minister level and 0 if he/she is at the director general level. The results are shown in columns (1) and (2) of Table 9. It can be found that the lower the university administrative level, the stronger the promotion of innovation by MOJIA. Such results may differ from existing studies^70^.

**Table 9:** Heterogeneity regression results.

	(1) Deputy Minister	(2) Director General	(3) “211”	(4) Non- “211”
	UI	UI	UI	UI
did	0.0192	0.2970***	0.1973***	0.3057***
	(0.179)	(4.717)	(2.672)	(4.005)
Rf	− 0.1853**	0.0278	− 0.1364**	0.0478
	(− 2.293)	(0.810)	(− 2.593)	(1.210)
Paper	− 0.0618	− 0.2092***	− 0.1312**	− 0.2135***
	(− 0.561)	(− 3.298)	(− 2.023)	(− 2.663)
Subject	− 0.1098	0.1006	0.1651*	0.0969
	(− 0.611)	(1.472)	(1.712)	(1.223)
Award	0.0704	0.0600	0.1217**	0.0324
	(0.751)	(1.586)	(2.227)	(0.727)
RDperson	− 0.1152	− 0.2537***	− 0.0790	− 0.3304***
	(− 0.811)	(− 3.503)	(− 0.765)	(− 3.930)
GDP	− 0.0794	2.1657***	− 0.6700	2.8049***
	(− 0.128)	(4.481)	(− 1.348)	(4.812)
Gov	− 0.2143*	0.3275***	0.2043**	0.2778**
	(− 1.841)	(3.786)	(2.389)	(2.550)
_cons	14.4108*	− 16.7201***	15.3315***	− 23.1860***
	(1.975)	(− 3.323)	(2.766)	(− 3.858)
University	Yes	Yes	Yes	Yes
Year	Yes	Yes	Yes	Yes
N	176	616	331	461
R^2^	0.8537	0.9284	0.8771	0.9227
Adj_R^2^	0.8197	0.9188	0.8566	0.9114
F	1.4265	10.2900	3.1034	9.1053

*t* statistics in parentheses.

*p < 0.1, **p < 0.05, ***p < 0.01.

According to resource dependence theory, organizations need access to external resources, especially those critical to their core business and competitive advantage, to achieve their goals and enhance their performance^43^. Universities at lower administrative levels may not have the same rich financial and reputational resources as top universities, and therefore rely more on their researchers' innovative capacity and knowledge output. MOJIA can incentivize researchers to continue innovating. This distribution of inventions' ownership can be seen as a resource allocation strategy that motivates researchers to innovate, thus increasing innovation output.

#### The heterogeneity analysis of university reputation

Next, we analyze the heterogeneity of the universities' social reputation. An organization's reputation determines how external evaluators perceive the products produced by the organization^70^. When information is asymmetric, technology demanders rely on intuition about evaluating university patents, that is, university reputation. Universities with high levels have cutting-edge research results highly attractive to industry^71^. Companies give a higher rating to the quality of universities' patents with social reputation, and under the influence of MOJIA, the UI should be further improved. There are many indicators to measure the universities' social reputation, such as "211" universities, "985" universities, and double-class universities. According to the principle that the grouping criteria should be exogenous, and considering the balance of the grouping samples, here we choose whether the universities are "211" universities to measure. The reason is that the "985" universities number is small, and the grouping lacks balance. The double-class universities were launched in 2017 and may be influenced by MOJIA without exogeneity. Therefore, we choose whether the university is a "211" to measure. The estimation results are shown in columns (3) and (4) of Table 9. It can be found that the MOJIA of non-"211" universities has a stronger effect on UI than "211" universities.

From the resource integration and optimization perspective, non-211 universities may focus more on in-depth cultivation and specialization in specific areas to maximize the use of limited resources. Researchers may concentrate their resources and efforts more on in-depth research, creating synergies in relatively small academic teams. This pooling and optimizing resources help improve research efficiency and quality, thereby increasing innovation output.

### The Moderating effect of intellectual property protection

The external innovation environment influences the innovation behavior of universities in addition to MOJIA. Since innovation activities have positive externalities^72^, universities will face innovation risk shocks if they lack a robust IPR protection system. As an essential innovation incentive system, IPP may affect the relationship between MOJIA and university innovation. Therefore, it is necessary to include it in the analytical framework. To examine the impact of IPP on the relationship between MOJIA and UI, a moderating effect model (5) is constructed in this paper. Among them, *IPP* is the level of intellectual property protection in the university's region. *k* indicates the province where the university is located. Suppose the regression coefficient *α*_*3*_ in the model (5) is significantly positive. In that case, it indicates that the higher the IPP level, the stronger the promotion effect of MOJIA on UI. However, we can see from column (1) of Table 10 that the coefficient of did*IPP is insignificant, and thus Hypothesis 2 is invalid.

5 $$UI_{it} = \alpha_{0} + \alpha_{1} \times did_{it} + \alpha_{2} \times IPP_{ikt} + \alpha_{3} \times did_{it} \times IPP_{ikt} + \alpha_{4} \times Controls_{it} + u_{i} + v_{t} + \varepsilon_{it}$$

**Table 10:** Mechanism analysis results.

	(1)	(2)	(3)
	UI	Tam	Tam
did	0.5305	1.6825***	1.6633***
	(1.548)	(5.956)	(5.795)
UI			0.0760
			(0.387)
IPP	0.0053		
	(0.008)		
did*IPP	− 0.3752		
	(− 0.825)		
Rf	0.0189	0.0454	0.0444
	(0.595)	(0.282)	(0.275)
Paper	− 0.2210***	0.2980	0.3151
	(− 3.908)	(1.023)	(1.069)
Subject	0.0905	0.0556	0.0486
	(1.460)	(0.173)	(0.151)
Award	0.0545	0.6358***	0.6313***
	(1.554)	(3.534)	(3.499)
RDperson	− 0.2127***	− 0.4735	− 0.4572
	(− 3.267)	(− 1.407)	(− 1.347)
GDP	1.8735***	3.3775	3.2274
	(4.313)	(1.579)	(1.484)
Gov	0.2327***	0.4550	0.4381
	(3.002)	(1.179)	(1.128)
_cons	− 12.7023***	− 29.8576	− 28.8218
	(− 2.751)	(− 1.324)	(− 1.269)
University	Yes	Yes	Yes
Year	Yes	Yes	Yes
N	792	792	792
R^2^	0.9358	0.8533	0.8533
Adj_R^2^	0.9275	0.8347	0.8345
F	8.2355	8.5044	7.5669

*t* statistics in parentheses.

*p < 0.1, **p < 0.05, ***p < 0.01.

### Economic consequence of UI

The conflict between the academic-oriented scientific research assessment system of universities and the market-oriented mechanism of enterprise technological innovation makes the gap between the R&D achievements of universities and market demand insurmountable, and the industrialization of university scientific research achievements faces the risk of "valley of death". With the implementation of MOJIA, the ownership of scientific and technological achievements is changed from being held by universities to being shared by universities and inventors. The ownership barrier to the universities' transformation of scientific and technological achievements no longer exists. Therefore, it is worth paying attention to whether universities can use effective institutional mechanisms, to cross the "last mile" of scientific and technological achievements transformation and improve the technical achievements marketability. Therefore, this paper will explore whether MAJIO can improve the regional technological achievements marketability by increasing UI. For this purpose, we constructed the following model for validation.

6 $$Tam_{kt} = \beta_{0} + \beta_{1} \times did_{it} + \beta_{2} \times Controls_{it} + u_{i} + v_{t} + \varepsilon_{it}$$

7 $$Tam_{kt} = \lambda_{0} + \lambda_{1} \times did_{it} + \lambda_{2} \times UI + \lambda_{3} \times Controls_{it} + u_{i} + v_{t} + \varepsilon_{it}$$

We know from the results above that MOJIA significantly promotes UI. When *β*_*1*_ and *λ*_*1*_ are significant, it indicates the presence of mediating effects. If *λ*_*2*_ is not significant, it indicates that there is a fully mediated effect of UI, otherwise it is a partially mediated effect. From the regression results in Table 10, it can be seen that the *did* coefficient in column (2) is significantly positive, indicating that MOJIA plays a significant role in promoting technology achievement marketability. In column (3), the *UI*'s coefficient is insignificant and the *did*'s coefficient is significantly positive, indicating that *UI* is fully mediated. That is, MOJIA can promote the technology achievement marketability by increasing *UI*. From the above results, we can find that MOJIA has a positive impact on improving *UI* and *Tam*. However, Chinese university science and technology achievements usually have problems such as substandard quality or far from market demand, and the transformation value of the achievements themselves is not high^73^.

From the viewpoint of obstructive factors of state-owned assets of scientific and technological achievements, unclear property rights among subjects and transaction costs are mainly caused by institutional factors. MOJIA is based on learning from extra-territorial experiences^74^, cracking the institutional dilemma through the allocation of rights, and enhancing the motivation of scientific researchers' technological transformation and their enthusiasm to buttress the technological needs of the market^75^. The participation of researchers in the transformation of achievements as civil subjects can eliminate the time and procedural costs brought by the state-owned assets management method^76^, thus improving the marketization of scientific and technological achievements. In MOJIA, the university's intellectual property institutional has been improved, and the incentive mechanism has been optimized, which makes teachers and researchers participate in innovation activities more actively and proactively. In addition, the reform has also prompted universities to change from traditional "academic thinking" to "market thinking", promoted the marketization and commercialization of regional technological achievements, and further improved the level of technological achievements marketization.

## Discussion and conclusions

### Discussion

Taking the data of MOJIA pilot universities and regions from 2012 to 2022 as a sample, this paper empirically analyzes the relationship and mechanism between MOJIA and university innovation (UI) in China by combining resource dependence theory and institutional theory. First, our study finds that MOJIA has a positive impact on UI. This conclusion is essentially in line with Kenney and Patton^77^. In short, it is not optimal for universities to maintain ownership of inventions, either from the perspective of economic efficiency or facilitating technology commercialization. Granting ownership to inventors can address issues such as university innovation and the marketability of technological achievements. From the existing research, some scholars have studied the factors influencing university innovation at the institutional level, such as the university Technology Transfer Office (TTO)^78,79^, industry support and interest^80,81^. There is no doubt that these factors are crucial for UI. However, these factors do not address ownership changes. This study investigates the mechanism of MOJIA on UI from the perspective of ownership change through a quasi-natural experiment. Therefore, this study is a valuable addition to the field of UI.

Second, the heterogeneity analysis shows that the promotion of UI by MOJIA is stronger in universities with lower administrative levels and “non-211” universities. In contrast to the conclusions of Hu et al.^70^, our study suggests that universities at lower administrative levels may not have the same rich financial and reputational resources as top universities, and therefore rely more on their researchers' innovative capacity and knowledge output. In some "non-211" universities, researchers may lack the incentive to engage in high-risk, high-reward inventions and innovations. Their achievements may not be adequately recognized and rewarded. Implementing MOJIA can motivate researchers to participate in innovation activities because they can share the benefits of their achievements, thus increasing motivation to innovate and improving the university innovation level.

Third, the study finds that MOJIA can promote Tam by increasing UI. Under the influence of MOJIA, by cooperating with enterprises, universities can better understand the market demand, transform inventions into actual products or services, and bring them to the market more quickly. For example, Southwest Jiaotong University gives researchers the right to own and use scientific and technological achievements in the long term. It has issued the Patent Management Regulations of Southwest Jiaotong University. This practice has transformed the current practice adopted by most universities of "transforming first and confirming rights later" into "confirming rights first and transforming later"^4^. MOJIA, therefore, makes university innovations more market-oriented. Universities can pay more attention to market demand and commercialization potential through cooperation with enterprises, which helps improve the market adaptability and competitiveness of university research results. Ultimately, it can improve the marketability of local technological achievements.

### Managerial and policy implications

First, improve the intellectual property system and optimize the management and operation mechanism. Efforts will be made to solve the inappropriate laws and regulations and related policies in implementing the current job invention empowerment process, improve the policy synergy effect, and create a good policy environment for the pilot program to be effective. Encourage the pilot units of job invention empowerment to strengthen the precise docking of policies, enhance the operability of pilot policies, and improve the flexibility of supporting policies. Granting researchers the freedom to assign their share of the invention patents they are entitled to, and enhancing the influence of researchers in the decision-making process of results transformation, to gain the honor of being able to serve the society by results transformation.

Second, improve the appraisal system of job inventions to encourage part-time innovation of scientific researchers. For university scientific researchers and applied talents who focus on the transformation of job inventions, different assessment indexes are formulated to fully mobilize the innovation consciousness and enthusiasm of university scientific researchers. Consider the amount of MOJIA comprehensively, and focus on the benefits of actual transformation of job inventions.

Thirdly, the university's administrative hierarchy, social reputation, and industrial background should be used wisely to promote the transformation of high-tech achievements of the university. The university formed these in the past and cannot be replicated, but it can only find another way. Universities without industry background and low administrative hierarchy can strengthen the construction of application-oriented good disciplines, start cooperation with leading industries and build future industrial research institutes. Universities lacking social reputations can target a particular advanced technology field for focused research. The competitive advantage of transforming scientific and technological achievements can be improved by strengthening the applied research capability.

### Limitations

Despite the theoretical and methodological strengths of this paper, it has limitations: First, due to the limitations of the database, this paper focuses on the impact of MOJIA on UI and the possible economic consequences. In further research, we can consider the impact of MOJIA on the efficiency of universities' technological achievements and the related boundary conditions. Second, future research could focus on the role that university top-management teams (TMTs) play in the relationship between MOJIA and UI—examining how the composition of TMTs affects the relationship between MOJIA and UI and the impact on the ownership of university researchers' inventions. For this purpose, drawing on the upper echelons theory and the attentional-based view is possible^82,83^. Third, we conducted our study in the singular context of China. While there are theoretical reasons to believe that universities in other countries may experience similar practices, namely, researchers are given ownership of their inventions achievements. However, focusing on a single country context may limit the study's results. Therefore, in the future, we can extract data from other developed or emerging economies and conduct empirical analyses using universities in other countries.
